# Human management and hybridization shape treegourd fruits in the Brazilian Amazon Basin

**DOI:** 10.1111/eva.12474

**Published:** 2017-05-04

**Authors:** Priscila Ambrósio Moreira, Cédric Mariac, Leila Zekraoui, Marie Couderc, Doriane Picanço Rodrigues, Charles R. Clement, Yves Vigouroux

**Affiliations:** ^1^ Post–Graduate Program in BotanyInstituto Nacional de Pesquisas da Amazônia (INPA)ManausAmazonasBrazil; ^2^ Institut de Recherche pour le DéveloppementUniversité de Montpellier (IRD)UMR DIADEMontpellierFrance; ^3^ Laboratório de Evolução AplicadaUniversidade Federal do Amazonas (UFAM)ManausAmazonasBrazil; ^4^ Coordenação de Tecnologia e InovaçãoINPAManausAmazonasBrazil

**Keywords:** agrobiodiversity, *Crescentia amazonica*, *Crescentia cujete*, introgression, plant domestication, wetlands

## Abstract

Local people's perceptions of cultivated and wild agrobiodiversity, as well as their management of hybridization are still understudied in Amazonia. Here we analyze domesticated treegourd (*Crescentia cujete*), whose versatile fruits have technological, symbolic, and medicinal uses. A wild relative (*C. amazonica*) of the cultivated species grows spontaneously in Amazonian flooded forests. We demonstrated, using whole chloroplast sequences and nuclear microsatellites, that the two species are strongly differentiated. Nonetheless, they hybridize readily throughout Amazonia and the proportions of admixture correlate with fruit size variation of cultivated trees. New morphotypes arise from hybridization, which are recognized by people and named as local varieties. Small hybrid fruits are used to make the important symbolic rattle (*maracá*), suggesting that management of hybrid trees is an ancient human practice in Amazonia. Effective conservation of Amazonian agrobiodiversity needs to incorporate this interaction between wild and cultivated populations that is managed by smallholder families. Beyond treegourd, our study clearly shows that hybridization plays an important role in tree crop phenotypic diversification and that the integration of molecular analyses and farmers’ perceptions of diversity help disentangle crop domestication history.

## 1 Introduction

Amazonia is an important center of plant domestication (Clement, 1999; Meyer, Duval, & Jensen, 2012). Its great biological and cultural diversity (Balée, 2013) make it an especially interesting area to study the role of human societies in plant domestication and diversification (Balée, 2013; Clement, de Cristo‐Araújo, D'Eeckenbrugge, Pereira, & Rodrigues, 2010). The distinction between wild and cultivated is one of the basic questions of plant domestication (Lévi‐Strauss, 1950; Pickersgill, 2013; Terrell et al., 2003). The distinction, however, often goes unnoticed, given the lack of understanding of how local people perceive biological diversity in traditional societies (Caillon & Degeorges, 2007). People's perceptions of cultivated and wild diversity, as well as their management practices that deal with plant hybridization, are still understudied in Amazonia, especially for tree species (Moreira, Lins, Dequigiovanni, Veasey, & Clement, 2015; Rollo et al., 2016; Smith & Fausto, 2016). Hybridization between related cultivated and wild plants may be favored or discouraged by local farmers (Jarvis & Hodgkin, 1999). It can promote domestication and diversification (Gompert & Buerkle, 2016; Miller & Gross, 2011), because hybrids often present interesting traits that can be selected and maintained (Goldschmidt, 2013; Miller & Gross, 2011; Zohary & Spiegel‐Roy, 1975), but may not be adaptive in natural environments (Ellstrand, 2003). This is especially true of introgressive hybrids, as backcrossing to one parent maintains its useful characteristics with minor influence of the other parent (Ellstrand, 2003; Harrison & Larson, 2014). It follows that hybridization and introgression between cultivated and wild plants, as well as the human practices that maintain diversity, are important for effective agrobiodiversity conservation. The distinction between wild and cultivated and its linkages with natural ecosystems are essential for a broader understanding of agriculture (Aumeeruddy‐Thomas, Hmimsa, Ater, & Khadari, 2014). More efforts are necessary for its recognition and implementation by public agricultural and forestry policies (Michon, Nasi, & Balent, 2013).

*Crescentia* spp. (Bignoniaceae) are excellent candidates to study hybridization and domestication associated with floodplains in Amazonia. *Crescentia cujete* Linnaeus (1753), known as treegourd or calabash tree, is an important tree crop for Amazonian smallholders (Lima & Saragoussi, 2000; Wittmann & Wittmann, 2011). Its versatile fruits, called *cuia* in Portuguese, are traditionally used as storage vessels, drinking cups, scoops to bail water from canoes, traps for fishing, diving masks, bags, body ornaments, ritualistic musical instruments, and, more recently, as “ecological” cups; they also have medicinal applications (Acostupa, Bardales, & Teco, 2013; Bennett, 1992; Bustamante, Hidalgo, & Frausin, 2011; Heiser, 1993; Morton, 1968; Patiño, 1967; Steward, 1948). *C. cujete* presents an ample variation in fruit shapes and sizes (Aguirre‐Dugua, Eguiarte, González‐Rodríguez, & Casas, 2012; Arango‐Ulloa, Bohorquez, Duque, & Maass, 2009; Gentry, 1980) that support the wide range of uses. A wild relative (*C. amazonica* Ducke 1937) occurs in flooded forests in the Orinoco and Amazon Basins, as well as smaller rivers of the Guianas (Díaz, 2009; Gentry, 1980; Godoy, Petts, & Salo, 1999; Wittmann et al., 2006). *Crescentia amazonica* fruits are smaller with thinner rinds that float in the water and are dispersed by fish (Waldhoff, Ulrich, & Furch, 1996).

The relationship between the two species is largely speculative. *Crescentia amazonica* was hypothesized to be the wild progenitor from which treegourd was domesticated (Ducke, 1946). Alternatively, treegourd was domesticated in Mesoamerica and later distributed to Amazonia (Gentry, 1980). In this case, it was hypothesized that *C. amazonica* was feralized *C. cujete* (Gentry, 1980). Their chloroplast genetic diversity does not support the possibility of *C. amazonica* being derived from *C. cujete* (Moreira et al., 2016). Nonetheless, a close relationship between *C. cujete* and *C. amazonica* is recognized by local human populations. In Guyana, people “called spirits” when *C. amazonica* was found, because they recognized it is a sort of “shadow” of the domesticated *C. cujete* (van Andel, 2000). As the two species co‐occur in Amazonia and *Crescentia* species are hypothesized to be largely interfertile (Gentry, 1980), gene flow might be abundant, but has not yet been shown at the molecular level.

In this study, we asked whether (i) hybridization plays a significant role in shaping genetic and morphologic diversity in *Crescentia* species, and whether (ii) hybrid and introgressed individuals are managed by Amazonian smallholders. To address these questions, we combined a genetic study based on chloroplast (single nucleotide polymorphisms—SNP) and nuclear (simple sequence repeats—SSR) markers with local farmer interviews. Using these datasets, we analyzed (i) the genetic differences between *C. amazonica* and *C. cujete* in the Amazon Basin, and gene flow between them; and (ii) the relationship between genetic and morphological diversity, and how people use and perceive this diversity.

## 2 Methods

### 2.1 Field sampling and interviews

**Figure 1:**
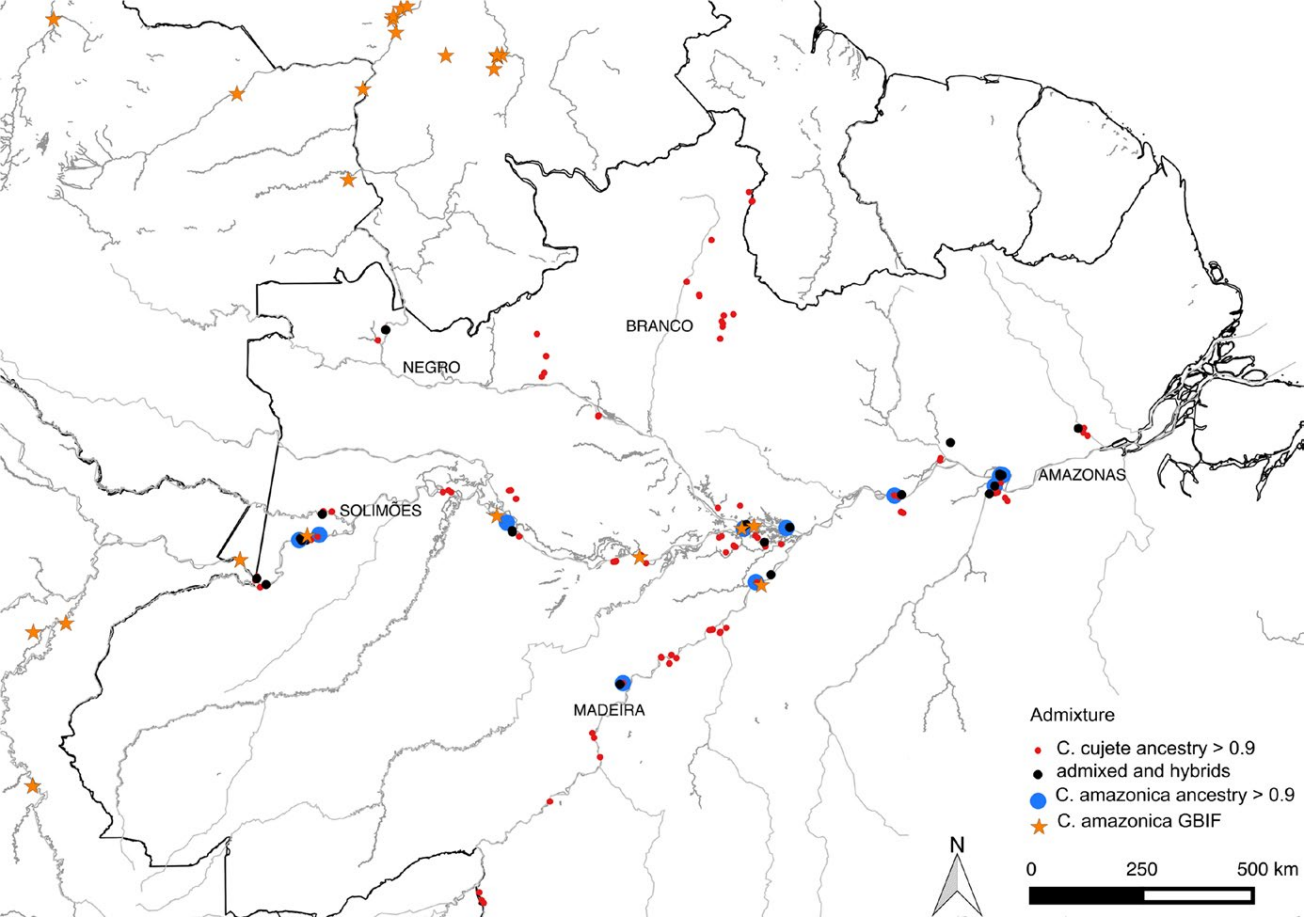
Geographical distribution of *Crescentia cujete* and *Crescentia amazonica* treegourds collected for this study along major rivers of Brazilian Amazonia (*N* = 234). Proportions of admixture identified by Structure at *K* = 2 are indicated. Proportions above 0.9 were considered pure and below 0.9 were classified as admixed. *Crescentia amazonica* records in northern South America are from the Global Biodiversity Information Facility

We visited rural and peri‐urban villages in 36 municipalities distributed along the major rivers of the Brazilian Amazon Basin (Figure 1). The broad geographical sampling followed two criteria for village selection: dependence on river resources and treegourd use in daily life. Data were collected after an informed consent invitation that was read collectively in each village and signed by a local representative. This research followed the International Society for Ethnobiology's code of ethics (International Society of Ethnobiology, 2006) and was approved by the Committee for Ethics in Research with Human Beings of the National Research Institute for Amazonia (CEP INPA, proc. no. 408.611, 2013). We collected leaves for genetic analyses of each treegourd (*N* = 469) found in domestic areas in the villages (Table S1). We considered domestic areas to include homegardens, swiddens, ports near the river, football fields, trails, and old homestead sites. Clones propagated from the same individual were avoided in order to better assess available diversity. Fruits were measured and photographed and their shape was categorized according to Arango‐Ulloa et al. (2009). We performed semi‐structured interviews about use, management, and history of most trees collected, and in nine municipalities we practiced participant observation of daily activities and urban farmers’ markets. To map wild treegourd distribution, we surveyed herbarium records of *C. amazonica*, and photographs of its fruit were presented to farmers in all villages visited to stimulate their memory of its presence in local flooded forests. We collected leaves of *C. amazonica* (*N* = 32) growing spontaneously in seven areas of flooded forests in six municipalities along the Solimões–Amazonas and lower Madeira rivers (Figure 1). Collection was authorized by the Brazilian System for Authorization and Information in Biodiversity, Chico Mendes Institute for Biodiversity Conservation, proc. no. 25052‐1, 2012, and transportation by the Brazilian Institute for the Environment and Renewable Natural Resources, proc. no. 14BR015576/DF, 2014.

### 2.2 Genetic analysis

DNA was extracted from dried leaves (*N* = 234) using the CTAB 5% protocol (Doyle & Doyle, 1990) with minor modifications. For nuclear SSR analysis, we genotyped all 234 samples, among which 184 were also analyzed for chloroplast SNPs (Table S1). Collected leaves without enough DNA or with low quality were excluded. To develop the nuclear SSRs, a barcoded library of *C. cujete* genomic DNA was sequenced (Moreira et al., 2016) using an Illumina MiSeqv.3 (San Diego, California, USA). QDD software 3.1.2 (Meglécz et al., 2009) was used to identify nuclear SSR motifs and design primers from 113,865 merged reads. The parameters used to select the nSSR primers were as follows: one primer pair for each read to avoid repeated regions of the genome; avoidance of mononucleotide microsatellites; preference for perfect microsatellites with ≥8 repeats; and avoidance of primers that are very close (≤20 bp) to the target SSR. A total of 1,436 SSR were identified, of which 1,068 were perfect SSR motifs with 819 di‐, 191 tri‐, 47 tetra‐, 10 penta‐, and 1 hexa‐repeat motifs, and 368 compound motifs. The primers were designed for the perfect SSRs (Table S2). We performed preliminary tests of amplification of 15 SSRs using *C. cujete* samples (*N* = 3). Five of the primer pairs failed to amplify, even using different temperatures and DNA concentrations, and were discarded. The remaining 10 SSR primers were labeled with fluorescence (FAM, NED, HEX; Applied Biosystems, Foster City, California, USA) and genotyped in an ABI 3130xL Genetic Analyzer (Applied Biosystems) using GS‐500 LIZ as the size standard (Applied Biosystems). Although all 10 SSR were polymorphic, at least for cultivated samples (*N* = 221), two loci were excluded (SSR2 and SSR9) because they failed to amplify in 60% of the samples. We kept the remaining eight SSRs (Table S3) for hybridization analysis, as they also amplified successfully for *C. amazonica*. Locus amplifications were made in simplex and multiplexed for fragment analysis, using the PCR kit (Qiagen, n.206143, Hilden, Germany) with the following program: 95°C for 15 min; 38 cycles, each of 94°C for 30 s, *T*_*a*_ °C for 1.30 min, and 72°C for 1 min; and a final step of 60°C for 30 min. Fragment size and allele identification were determined using GeneMapper (Applied Biosystems). We also obtained SNPs observed in the whole chloroplast using previously described approaches (Moreira et al., 2016; Scarcelli et al., 2016).

### 2.3 Diversity analyses

The eight nuclear SSRs were used to genotype 234 treegourds, among which 221 were from domestic areas and 13 from flooded forests (Table 1). Relationships among individuals were assessed with Structure 2.3, using the admixture model (Pritchard, Stephens, & Donnelly, 2000). We varied the number of genetic clusters (*K*) from *K* = 1 to 20, with 100.000 burn‐in, 100.000 iterations, and five different runs for each *K* value. The ad hoc ΔK (Evanno, Regnaut, & Goudet, 2005) was used to identify the most likely number of clusters in the matrix. We considered an individual to belong to a given cluster if its proportion of admixture was <0.10, that is, with more than 0.90 of the individual's SSR profile attributable to the given cluster. In this study, we used the term “admixed” for ancestry between 0.90 and 0.60, and the term “hybrid” for ancestry from 0.60 to 0.40 (Table S1). We also calculated a hybridization index using Introgress 1.2.3 (Gompert & Buerkle, 2010) and compared it with the Structure admixture proportions. The whole chloroplast sequences of 174 domestic treegourds and 10 from flooded forests were analyzed (Table S1). We built a haplotype network based on 250 SNPs using the median joining algorithm (Bandelt, Forster, & Röhl, 1999). The network was visualized using the samples with <4% of missing data, according to software requirements in POPART 1.7 (Leigh & Bryant, 2015). The nuclear and chloroplast comparison defined paternal and maternal introgression, respectively, and determined the final botanical identification (170 *C. cujete* and 14 *C. amazonica*). To assess the impact of the uneven number of samples of the two species, we performed a complementary Structure analysis using the same sample size for both species (14 *C. amazonica*, 14 *C. cujete*; Fig. S1), with the *C. cujete* samples chosen at random. Finally, we assessed the potential impact of null alleles on admixture inferences (Fig. S2), by coding any missing data as a homozygote recessive allele (Falush, Stephens, & Pritchard, 2007).

**Table 1:** Summary of nuclear (SSR) admixture proportions (*N* = 234) and chloroplast (SNPs) haplotypes (*N* = 184) of the *Crescentia cujete* and *Crescentia amazonica* collections analyzed in this study and the habitats they were collected in. A. Admixture proportions for *C. cujete* and *C. amazonica* in columns and chloroplast haplotypes in lines. Not confirmed means the chloroplast was not analyzed in plants that were genotyped with nSSR. B. Habitats in which pure and admixed treegourds were collected

			Pure cujete	Cujete admixed	Hybrids	Amazonica admixed	Pure amazonica
	nSSR (*N* = 234)	*N*	175	25	11	5	18
A)							
Haplotypes (*N* = 184)	*C. cujete*	170	142	16	8	1	3
	*C. amazonica*	14	0	0	1	2	11^a^
	Not confirmed	50	33	9	2	2	4
B)							
Domestic areas (*N* = 221)			175	25	11	5	5
Flooded forest (*N* = 13)			0	0	0	0	13^b^

a *Crescentia amazonica* with “pure amazonica” assignment in Structure at *K* = 2 is predominant in flooded forests (*N* = 10), but one was found cultivated (*N* = 1). Admixed *C. amazonica* were only found in domestic areas.

b All of these are likely to be *C. amazonica*, but three were not analyzed for their chloroplast haplotypes.

Nuclear genetic diversity of *C. cujete* and *C. amazonica* was explored with principal components analysis (PCA) executed with stats R package (R Core Team, 2015). Nuclear genetic diversity and species differentiation were estimated using hierfstat (Goudet, 2005). Pairwise *F*_ST_ were calculated and statistically assessed using 1,000 bootstraps (Nei, 1987). The significance of *F*_*IS*_ was measured as deviation from Hardy–Weinberg equilibrium using pegas R (Paradis, 2010). Chloroplast diversity was estimated using DNAsp 5.10.1 (Librado & Rozas, 2009) and Arlequin 3.5 (Excoffier & Lischer, 2010). We examined the relationship between *C. amazonica* admixture proportions and *C. cujete* fruit diameters (*N* = 61) with simple regression in R package (R Core Team, 2015).

## 3 Results

### 3.1 Genetic structure revealed with nuclear SSRs

**Figure 2:**
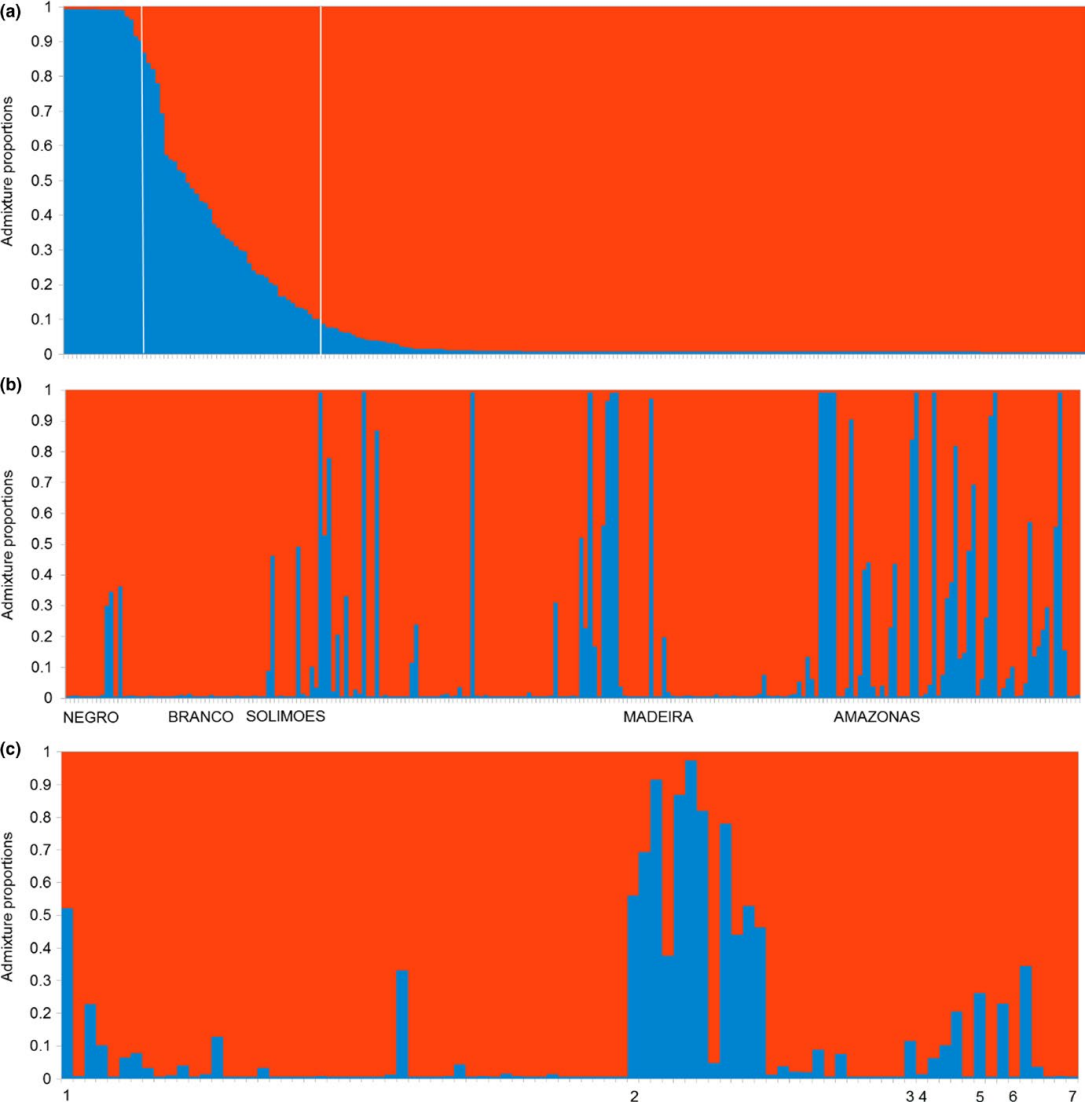
Structure analysis of 234 treegourd samples collected in Brazilian Amazonia. The *y*‐axis shows the proportion of assignment to the groups at *K* = 2 (red—*Crescentia cujete* and blue—*Crescentia amazonica*). (a) Samples are ordered by their proportion of admixture: admixed if >0.1, hybrids if 0.4 to 0.6, pure if >0.9 of assignment to the group. (b) Samples were ordered by their geographical location along the main rivers: the Negro, Solimões, and Amazonas rivers are ordered west to east; the Branco River is ordered north to south; the Madeira River is ordered south to north. (c) Samples are ordered by seven fruit shapes (see Figure 4) and fruit size, with size increasing from left to right

The Structure analyses identified two clusters as the most likely structure (*K* = 2, Figure 2a), based on the ad hoc ∆*K* approach (Evanno et al., 2005; Fig. S1). The two clusters correspond to domesticated *C. cujete* and wild *C. amazonica*, the two accepted botanical species already described in the Amazon Basin. We also found a significant amount of admixture between them (Figure 2a, Table 1), especially along the Solimões–Amazonas River (Figures 1 and 2b). The frequency of individuals with admixture proportions suggesting hybridization and introgression was similar in both groups. Among the 234 samples, 200 were assigned primarily to *C. cujete*, of which 24 (12%) were admixed to some degree, and 23 were assigned to *C. amazonica*, of which five (21%) were admixed (Table 1). Eleven plants presented admixture proportions between 40% and 60% and were classified as hybrids. These proportions were similar when the *C. cujete* sample size was reduced at random to be equal to the *C. amazonica* sample size (*r* ^2^ = .99, *p *<* *10^−15^, Fig. S1). At the second possible grouping (*K* = 3, Fig. S3), similar admixture proportions were also found (*r* ^2^ = .98, *p *<* *10^−15^), while the original *C. cujete* group was subdivided without relation to geography or fruit morphology (Fig. S3). The hybridization index calculated with Introgress was highly correlated with the admixture proportions calculated with Structure (Fig. S4, *r* ^2^ = .83, *p *<* *10^−15^). Finally, coding all missing data as homozygous recessive alleles did not have an impact on admixture inferences (Fig. S2, *r* ^2^ = .96, *p *<* *10^−15^).

### 3.2 Comparison between chloroplast SNPs and nuclear SSR diversity

**Figure 3:**
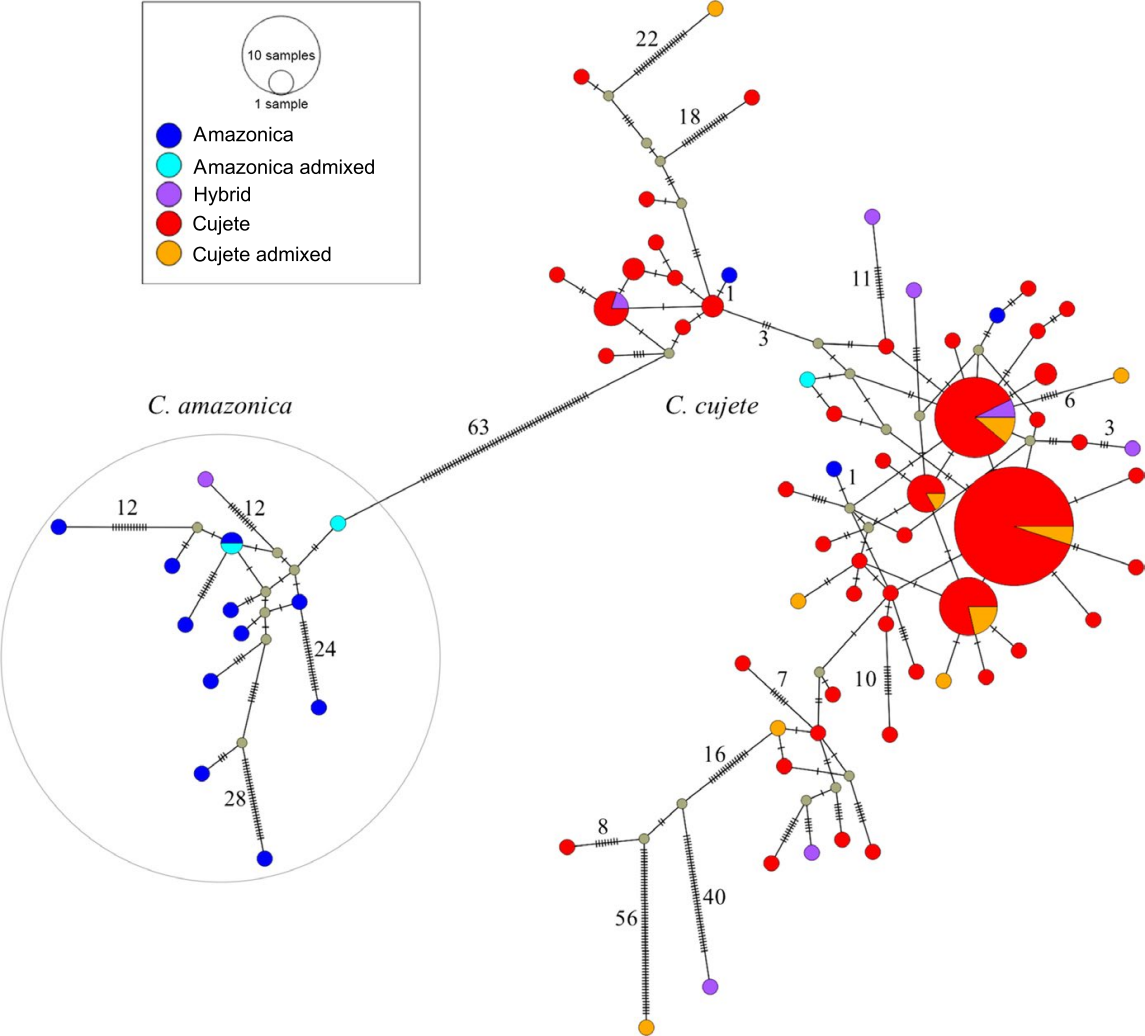
Chloroplast haplotype median joining network of *Crescentia cujete* and *Crescentia amazonica* from Brazilian Amazonia based on 250 chloroplast SNPs. Nuclear ancestry of each sample (*N* = 184) was evaluated using Structure (Table 1). The size of the circle reflects the number of individuals presenting the same haplotype. Numbers of mutations are indicated as hatch marks and numbers between haplotypes. Colors represent percentage of nuclear admixture, where *C. amazonica* >0.9 (dark blue); admixed *C. amazonica* <0.9 and >0.6 (light blue); hybrids <0.6 and >0.4 (violet); admixed *C. cujete* <0.9 and >0.6 (orange); and *C. cujete* >0.9 (red). A clear chloroplast difference is observed between *C. amazonica* and *C. cujete*, although 17% (*N* = 4) of the *C. amazonica* ancestry samples (*N* = 23, Table 1) have *C. cujete* haplotypes

The chloroplast analyses also clearly identified the two botanical species (Figure 3), with 63 mutational differences between the *C. cujete* and *C. amazonica* chloroplast sequences. In the haplotype network, samples with *C. cujete* nuclear assignments showed exclusively *C. cujete* chloroplast haplotypes (Figure 3, Table 1). For individuals with *C. amazonica* nuclear assignments (*N* = 23), 77% had *C. amazonica* haplotypes (*N* = 13) and 17% had *C. cujete* haplotypes (*N* = 4). Among the hybrids, most had *C. cujete* haplotypes (88%, *N* = 8), and one had a *C. amazonica* haplotype. The flooded forests harbored exclusively pure *C. amazonica* samples (*N* = 10), while domestic areas harbored pure and admixed samples of both species (Table 1, Figure 5c). The genetic diversities of the pure samples of both species were lower than their admixed samples (Table S4). The rarified allele count (*A*_*r*_) varied from 1.1 to 5.4, with the lowest value in *C. amazonica*, slightly higher within its admixed samples and the highest value in admixed *C. cujete*. The mean expected heterozygosity (*H*_*s*_) of pure *C. cujete* was 0.31 and that of pure *C. amazonica* was 0.09, while their admixed samples showed mean *H*_*s*_ values of 0.58 and 0.33, respectively (Table S4). *C. amazonica* showed extremely low diversity, with fixed alleles at six loci. These low diversity values for *C. amazonica* are probably due to marker development from *C. cujete* with consequent poor transferal due to the significant divergence between species. Although such bias might lead to imprecise estimation of *C. amazonica* diversity, it does not have an impact on the identification of hybrids, as admixture is based on allele frequency differences and not diversity per se (Pritchard et al., 2000).

Differentiation between *C. cujete* and *C. amazonica* was very high (*F*_ST_ = 0.74, IC_95%_ = 0.59–0.80 excluding admixed samples; *F*_ST_ = 0.56, IC_95%_ = 0.41**–**0.66 with all samples). *F*_*IS*_ (Table S4) was not significant for *C. cujete*, but significant for *C. amazonica* (*F*_*IS*_ = 0.44, *p *<* *.05). In the chloroplasts, we found 92 SNPs in 14 individuals of *C. amazonica* with 14 haplotypes, and 178 SNPs in 170 individuals of *C. cujete* with 71 haplotypes. Nucleotide diversity (π) was 3 × 10^−3^ and 1.2 × 10^−3^, for *C. amazonica* and *C. cujete*, respectively. Chloroplast differentiation between species was high (*F*_ST_ = 0.89, *p *<* *.05).

### 3.3 Admixture proportions correlated with fruit size

**Figure 4:**
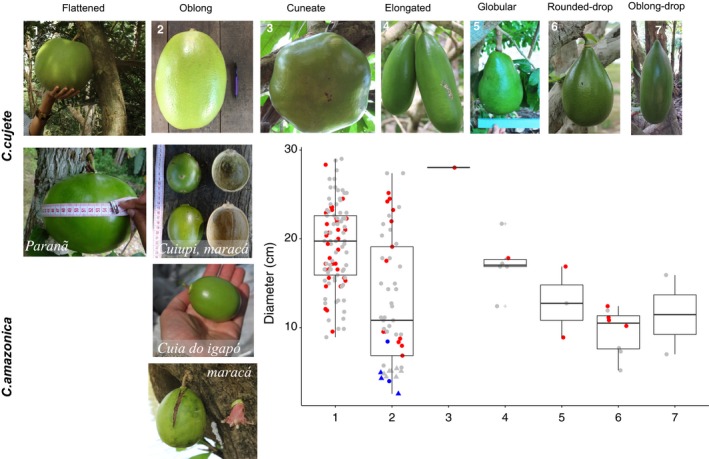
Diversity of fruit shapes and sizes in Amazonian treegourds. Seven fruit shapes of *Crescentia cujete* and one shape of *Crescentia amazonica* were found in the Brazilian Amazon Basin. Shape classification follows Arango‐Ulloa et al. (2009). Smaller fruits of types 1 and 2 have local names that are shown in italics in the corresponding photograph. Box plots of the variation in diameter (cm) of each fruit shape, with domestic (●) and wild individuals (▲). The colors represent chloroplast haplotypes (red—*C. cujete*; blue—*C. amazonica*; gray—not confirmed). In fruit shape 2, fruits smaller than 10 cm can be *C. amazonica* or admixed *C. cujete*, in which case they correspond to the *maraca* fruit type

**Figure 5:**
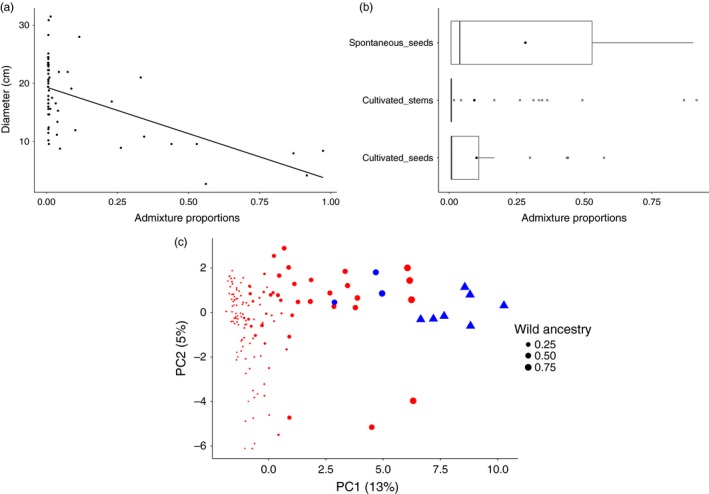
Admixture proportions of treegourds based on eight nSSR and its relationship with fruit size and propagation method, and the dispersal of diversity in the principal component analysis. (a) Fruit diameter of genetically confirmed *Crescentia cujete* samples (*N* = 61) as a function of the proportion of admixture with *Crescentia amazonica*, where diameter = 17.9–2.07*admixture (*r* ^2^ = .34, *p *=* *1.26 × 10^−6^). (b) Admixture proportions of *C. cujete* (*N* = 80) samples propagated spontaneously by seeds and cultivated by stems and by seeds. Black dots indicate the average size and white circles are outliers that highlight the active maintenance of admixed individuals by people. (c) Principal components analysis of the genetic relationships among *Crescentia cujete* (*N* = 170; red (●)) and *C. amazonica* (*N* = 14; blue (▲)) individuals. The proportion of the variance explained by each PC is shown in parentheses along each axis. The gradient of admixture is visible along PC1 and the admixed individuals correspond to the smaller fruit size varieties *paranã* (flattened type), *maracá,* and *cuiupi* (oblong types) found exclusively in domestic areas

There is ample morphological diversity of treegourd fruits in the Brazilian Amazon Basin (Figure 4). We recorded seven types of *C. cujete* fruit shapes in domestic areas (*N* = 167) and one fruit type of *C. amazonica* in flooded forests (*N* = 10). In domestic areas, two of them account for 86.4% of the plants: 63% flattened (type 1) and 23.4% oblong (type 2). The other five types were rare: 0.6% was cuneate (type 3), 3.6% elongated (type 4), 1.8% globular (type 5), 4.8% rounded‐drop (type 6), and 1.2% oblong‐drop (type 7). The two *C. amazonica* samples found in domestic areas showed type 2 fruits. While the fruits of *C. amazonica* in the flooded forest have small diameters ranging from 2.5 to 5.4 cm (median: 4.85 cm), in domestic areas diameters were slightly larger, ranging from 4 to 8.4 cm (median: 6.2 cm) (Figure 4). Among *C. cujete*, there is great size variation, especially within types 1, 2, and 5, with variation from 5.2 to 29 cm. Smaller fruits of *C. cujete* were correlated with *C. amazonica* admixture (*r* ^2^ = .34, *p *=* *1.26 × 10^−6^, Figure 5), especially the oblong (type 2) fruit (*r* ^2^ = .62, *p *=* *4.81 × 10^−6^). This relation remains even if we exclude the most extreme admixed samples (wild ancestry higher than 0.8, *r* ^2^ = .23, *p *=* *1.3 × 10^−4^). The smaller *C. cujete* fruits, between 3 and 10 cm in diameter, are those with the highest admixture proportions (Figure 5). Some *C. amazonica* ancestry could be observed in all shapes found in domestic areas, except in the rare type 7 (Figure 2c).

### 3.4 Use, management and perception of fruit diversity

We recorded 11 current domestic uses of *C. cujete* fruits that are related to fruit morphology and were categorized in levels of specialization. Five uses are related with fruit size, without restriction of shape. The small gourds that are mainly used for food consumption (*xibé*, a meal of water and manioc flour), *açaí* (juice from *Euterpe oleracea* or *E. precatoria*), water, or some particular medicine were reported in 31% of the municipalities. In the upper Negro River, these are called *cuiupi*. Large gourds are preferred for use as baskets to store seeds, seedlings, and cleaning products or to serve food (fish, *tapioca*), and were reported in 66% of the municipalities. Large gourds are also used as a unit of measurement of manioc during the preparation of flour and were reported in 47% of the municipalities, for the daily bath in the river (39%), to bail water from inside the canoe (39%), and pulp of broken fallen fruits as fodder for livestock (16%).

Four uses require greater specialization because they need a combination of size and fruit shape. Larger (20–24 cm) oblong fruits (type 2) are used to make a kind of bag with fiber handles (called *coió*,* comboró*,* mocó*), recorded in 30% of the municipalities, and used to transport things to the swiddens or to fish. Flattened fruits (type 1) with relatively smaller sizes (13–15 cm) are used in different contexts. They are involved in *tacacá* commerce, a typical Amazonian soup served in treegourd bowls (called *paranã*), whose manufacture was recorded in 11% of the municipalities. They are also used to make bowls to prepare blessings (14 cm) during religious events, recorded in 8% of the municipalities, and more rarely as parts of clothing sewn for regional festivals (2.8% of the municipalities).

Finally, there is an opportunistic use of fruit diversity, recorded in 16% of the municipalities, which incorporates different fruit shapes and sizes into the repertoire of manufactured objects sold as handicrafts. In this repertoire, even the wild species, *C. amazonica,* with its small fruits*,* is included, as recorded in four municipalities along the Solimões–Amazonas River. Its fruits are designed as small cups, painted and sold to cosmetic and food companies as “ecological cups,” and are worth U$ 6 per 100 cups for handicrafters (price in 2014). Other wild fruit type handicraft designs include the reproduction of ancient musical instruments called *maracá*, and also as toys for children (*carrapeta*). The use of these artifacts was recorded in domestic contexts in 8% of the municipalities, all along the Solimões–Amazonas River, where admixture is relatively frequent and with higher levels (Figure 2b).

While large fruits have a general name (*cuia* and *coité*) with variations that include shape information (e.g., long gourd), the smaller fruits are distinguished with different local names that are related to flooded environments: *cuia do igapó*,* cuiupi*,* paranã,* and *maracá. “Cuia do igapó”* is the wild tree that grows spontaneously in the *igapó,* a local word for a forest environment that is periodically flooded by black or clear water (Irion, Junk, & Mello, 1997), and has smaller size fruit (*“it is the same as cuia, but is small, is from the forest, is not planted”*). The other smaller gourds mentioned depend on human intervention to survive and are found exclusively in domestic contexts. *Cuiupi* was cited along the Madeira River as similar to the wild type (“*there is cuiupi in the lakes, near the river, but I prefer the big one”*), while along the upper Negro River it is the general term for small gourds. *Paranã* is a local word to describe secondary river channels, often between the edge of the forest and the white‐water floodplain system rich in nutrients and sediments (Irion et al., 1997), the habitat of *C. amazonica*. *Maracá* develops increased fruit size in domestic contexts (“*maracá is the cuia do igapó, but it is bigger”*) and its fruit production is restricted to flooding season (“*the fruit fails, does not give all the time*”). Based on genetic analyses and people's designation of varieties, *cuia do igapó* refers to *C. amazonica,* while *cuiupi* and *paranã* are *C. cujete* with moderate admixture proportions. *Maracá* is a special case, as it is applied not only to admixed *C. cujete*, but also to *C. amazonica*.

Cultivation by seeds, but also by stems, maintains highly admixed samples (Figure 5b), as many desirable gourds have smaller sizes, which is partly due to admixture effects (Figure 2c). Most treegourds are propagated by stem cuttings (61% of the sampled trees), and less frequently by seeds (37%). Stem cuttings have a purpose: They ensure a fast way to produce fruits (“*by seeds takes more time*”) and the maintenance of fruit morphology, avoiding those fruits that break easily (“*by stems it is better for the cuiupi not to become soft and break*”). This is also the preferred method among traders who cultivate large clonal areas in upland environments for *tacacá* bowls, the flattened smaller gourd (type 1) with moderate levels of admixture called *paranã*. Seed propagation can be a spontaneous event when flood water brings seeds (28%) or spontaneously propagated through discarded fruit pulp near the house (71%). Seedlings, despite the lack of guarantee in fruit morphology, can survive floods better than cuttings in floodplain landscapes. People stated that: *“it is not correct to plant seeds, but stems do not take here,*” *“when it grows by seeds, from the pulp, it grows smaller.”* In the floodplain, stem cuttings require greater effort (“*to make big cuia takes much work*”), and success is not guaranteed (“*it is not all the stems that work, here I've tried hard*”). Thus, seed propagation is a way to deal with the high annual flooding events in the floodplain. Another motivation is that seedlings are more suitable to produce smaller fruit sizes that are also useful, especially for handicraft purposes, as observed in the social movement of craftswomen along the middle Amazonas River.

## 4 Discussion

### 4.1 Identification of geographically widespread admixture

The tenuous nature of reproductive barriers among *Crescentia* species (Gentry, 1980) was confirmed by the large amount of admixture observed between *C. cujete* and *C. amazonica*. Our inference of admixture proportions was very robust even with unequal sample sizes and was correlated with the hybridization index of Gompert and Buerkle (2010), even with our small set of nuclear makers. The main reason is certainly the very high differentiation between the two species (*F*_ST_ = 0.74 with nSSR; *F*_ST_ = 0.89 with cSNP). Although we could not rule out the existence of null nSSR alleles in *C. amazonica*, they do not influence the high differentiation observed between species. Different histories of gene flow might result in similar patterns of admixture proportions (Barton & Hewitt, 1985; Gompert & Buerkle, 2016). In this case, one question is whether these admixture proportions reveal hybridization after secondary contact or a long‐term divergence. The high number of substitutions between *C. cujete* and *C. amazonica* chloroplast sequences suggests ancient divergence between the two species. As *C. cujete* is a species with cultivated populations, the origin of this domestication is unlikely to be older than other domestications in the Americas, which started by 11,000 years (Piperno, 2011). The chloroplast sequence divergence that we found suggests that the two species diverged earlier. Therefore, *C. cujete* was likely introduced by humans into South America (Gentry, 1980), and the admixture observed is secondary contact.

The hybrids are concentrated along the east–west axis of the Amazon Basin, the Solimões and Amazonas rivers (Figure 1). Although proximity to floodplains is an important parameter for the occurrence of hybrids, admixed individuals are also found beyond *C. amazonica*'s known distribution. One example is the occurrence of admixture along the Negro River, where no *C. amazonica* has been collected to date (Figures 1 and 2b). This pattern might result from social networks and propagule exchange of admixed plants by humans between rivers, such as between the Orinoco and Negro basins (Hornborg, 2005; Lathrap, 2010).

### 4.2 Flooded forests are a source for cultivated treegourd phenotypic diversity

Fruit size variation of domesticated *C. cujete* in Amazonia is partly shaped by admixture between wild and cultivated plants. Note here that we do not have a common garden experiment to evaluate the fruit phenotype. Establishing such a common garden will be difficult because treegourd is of minor economic importance outside local communities, and consequently, there is no conservation or breeding in a research institution to allow working in already available common gardens. However, most of the cultivated plants measured shared a cultivated environment in homegardens. If size was simply associated with variability of the environment, we would not detect a significant association with introgression. The absence of a common environment certainly adds more variability in size, but consequently also renders significant correlations with admixture more difficult to detect.

As larger fruit size is an expected feature of tree domestication syndromes (Meyer et al., 2012; Miller & Gross, 2011), hybridization and introgression create variation in fruit size (Cornille, Giraud, Smulders, Roldán‐Ruiz, & Gladieux, 2014) that can be managed (Aumeeruddy‐Thomas et al., 2014; Cornille et al., 2012; García‐Marin, Hernández‐Xolocotzi, & Castillo, 1986; Hughes et al., 2007; Zerega, Ragone, & Motley, 2004). The great diversity of fruit sizes in Amazonian homegardens was also observed in the floodplains of the Orinoco River and the Caribbean regions of Colombia (Arango‐Ulloa et al., 2009). Similarly, in the Yucatán Peninsula of Mexico, large propagated fruits and spontaneous smaller fruits were reported in homegardens (Aguirre‐Dugua et al., 2012). This suggests that perceptions of hybridization are used to manage fruit size and shape across the Neotropics.

Our results showed that pollen gene flow occurs in both directions between these *Crescentia* species. Bat pollination observed in both species (Fleming, Geiselman, & Kress, 2009) certainly favors this pollen flow between villages and flooded forests. However, the hybrid plants were restricted to human‐managed areas. Hybrid and introgressed *C. cujete*/*C. amazonica* are certainly selected against in the flooded forest and favored in human areas, as adaptation to natural environments is likely to be reduced by hybridization with domesticated populations (Ellstrand, 2003).

### 4.3 Traditional communities manage hybridization

Use of larger treegourds is widely distributed throughout the Amazon Basin, while the use of smaller ones is reported in less than half of the municipalities visited. Small fruits are much appreciated for the manufacture of handicrafts. However, the record of domestic artifacts made of small fruits suggests ancient use and not only contemporary commercial handicraft demand. The historical use of smaller treegourd fruits was mentioned by many ethnographers, especially in rituals and for medicinal purposes. *Maracá* is a symbol in spiritual practices of different Native Amazonian cultures, as well as an ancient rattle, and *paranã*, the flattened round type, was traditionally used for bowls in ceremonial rituals (Lévi‐Strauss, 2004; Ribeiro, 1995; Steward, 1948). Both of these objects made from smaller treegourds are considered by oral histories as the first things to be in the world in different cultures of Mesoamerica, the Antilles, and South America (Heiser, 1993), such as the Taíno from the Dominican Republic (Martin, 1999), the Guarani from southern Brazil (Montardo, 2002), and the Tukano from Amazonian Brazil and Colombia (Hugh‐Jones, 2009). These smaller fruits come from admixed individuals (Figures 4 and 5), which are maintained and dispersed by people. In the Brazilian Amazon Basin, they are recognized as local varieties (*cuiupi*,* paranã*,* maracá,* and *cuia do igapó*), whose names and associated traditional ecological knowledge are explicitly related to *C. amazonica* habitat, or wild‐cultivated hybridization and its morphological consequences. The association with flooded environments is also present in the Tupi origin of the name *cuiupi* (from *kuy’ y*) that refers to gourds of the water (Ferreira, 2004). The name *maracá* (*mbara'ka*) refers to the small rattles played in order to talk to lakes and heal sick people (Andía, 2015). *Maracá* was also used by Ducke (1946) when he described *C. amazonica* collected in flooded forests of the Solimões River in 1937. All of this confirms that the small fruited varieties used to manufacture important objects in the Amazon Basin, such as *maracás*, are the result of human selection of hybrid and admixed trees, and highlight that treegourd diversity is partly dependent on hybridization between homegarden and flooded forest genepools.

Although there is an historical use of these admixed treegourds, there is no evidence of ancient cultivation of *C. amazonica*. Our documentation of cultivation of *C. amazonica* along the Solimões–Amazonas River, especially in Santarém handicrafter villages, appears to be a recent practice. Treegourd handicrafts have been famous since before the Colonial period (Medina, 1934; Patiño, 1967; Rodrigues Ferreira, 1933) and gained prominence recently as a Brazilian Cultural Heritage (IPHAN, 2015). Handicrafts are motivated not only by social and economic demands, as highlighted by Santos (1982) and Carvalho (2011), but also ecological pressures, as severe flooding in these areas influenced people to use seedlings as an alternative way to produce treegourds for sale. As a result, we observed a high frequency of admixture in these handicrafter villages located in the middle Amazonas River (Figure 2b). The most common cultivation practice of *C. cujete,* however, is vegetative propagation, not only in Amazonia, but also in Mexico (Aguirre‐Dugua et al., 2012). This is the traditional way to maintain useful fruit phenotypes, a practice that allows management of hybridization also (Miller & Gross, 2011). The admixed treegourds have been dispersed by humans along Amazonian rivers and potentially over larger geographical areas, which might create a complex pattern of geographical admixture, as observed for several other Neotropical fruit trees and their wild populations [*Spondias* (Miller, 2008); *Inga* (Dawson et al., 2008); *Chrysophyllum* (Petersen, Parker, & Potter, 2014)], as well as in the Old World genus *Prunus* (Delplancke et al., 2012). Therefore, human activity not only maintains, but promotes congener interaction, as expected with other crops (Anderson, 2005; Riesenberg & Wendel, 1993). Hybrids are perceived and propagated in a dynamic way, so that hybridization is managed according to people's needs. This is in agreement with local farmers’ practices and experimentation observed worldwide (Brush, 2000; García‐Marin et al., 1986; Hughes et al., 2007; Jarvis & Hodgkin, 1999).

## 5 Conclusions and perspectives

We provided evidence that variation in fruit size of the two *Crescentia* species found in the Brazilian Amazon Basin is related to their admixture proportions. New morphotypes that arise from hybridization are clearly recognized by people and named as local varieties (*maracá, cuiupi, paranã*), whose symbolism is emblematic for Amazonian cultures. Beyond treegourd, our study clearly shows that hybridization plays an important role in crop phenotypic diversification. We also showed that the integration of molecular analyses and farmers’ perceptions of diversity can help disentangle crop domestication history. The specific traditional uses suggest that admixture management is an ancient human practice, also used in current traditional communities. We found that treegourd phenotype diversity depends partially on gene flow between homegardens and flooded forests. These results highlight the linkages between agriculture and forest ecosystems necessary for effective conservation of Amazonian agrobiodiversity. This is especially important as traditional ecological knowledge and floodplain conservation are neglected by development models for Amazonia (Castello et al., 2013; Posey & Balick, 2006).

## Data sharing

1. Geographical coordinates are available in Table S1.
2. Vouchers of *C. amazonica* (Lat −4.32/Lon −59.71 and Lat −2.11/Lon −54.72) were deposited in the National Research Institute of Amazonia Herbarium (numbers 255.829 and 266.725).
3. The sequence alignments and microsatellite genotypes are available in Dryad doi: https://doi.org/10.5061/dryad.t84p3
.

## Author contributions

PAM, CRC, and YV planned the study. PAM carried out the field collections and interviews. PAM, LZ, MC, and DPR performed the molecular work. PAM, YV, and CM carried out the genetic analysis. PAM, CRC, and YV wrote the manuscript.
