# Supplementary figures and images for: Human management and hybridization shape treegourd fruits in the Brazilian Amazon Basin
Source: Evol Appl. 2017 May 4;10(6):577–89. doi: 10.1111/eva.12474 (PMC5469164; doi:10.1111/eva.12474)

Total sampling

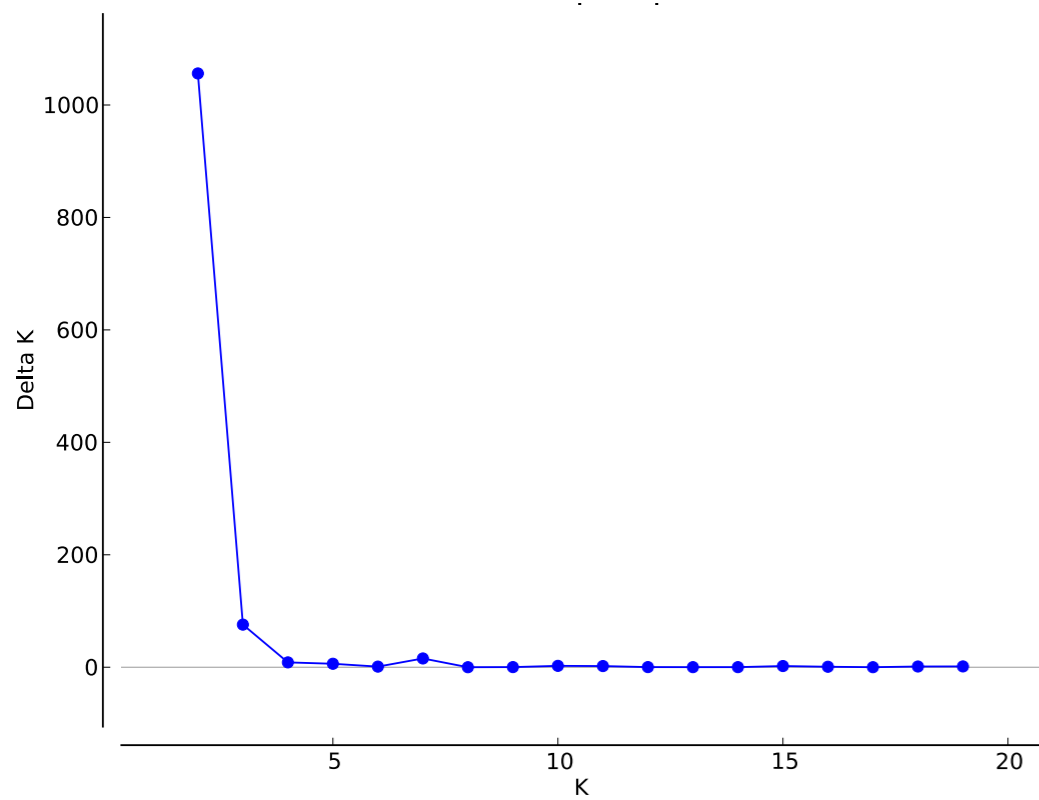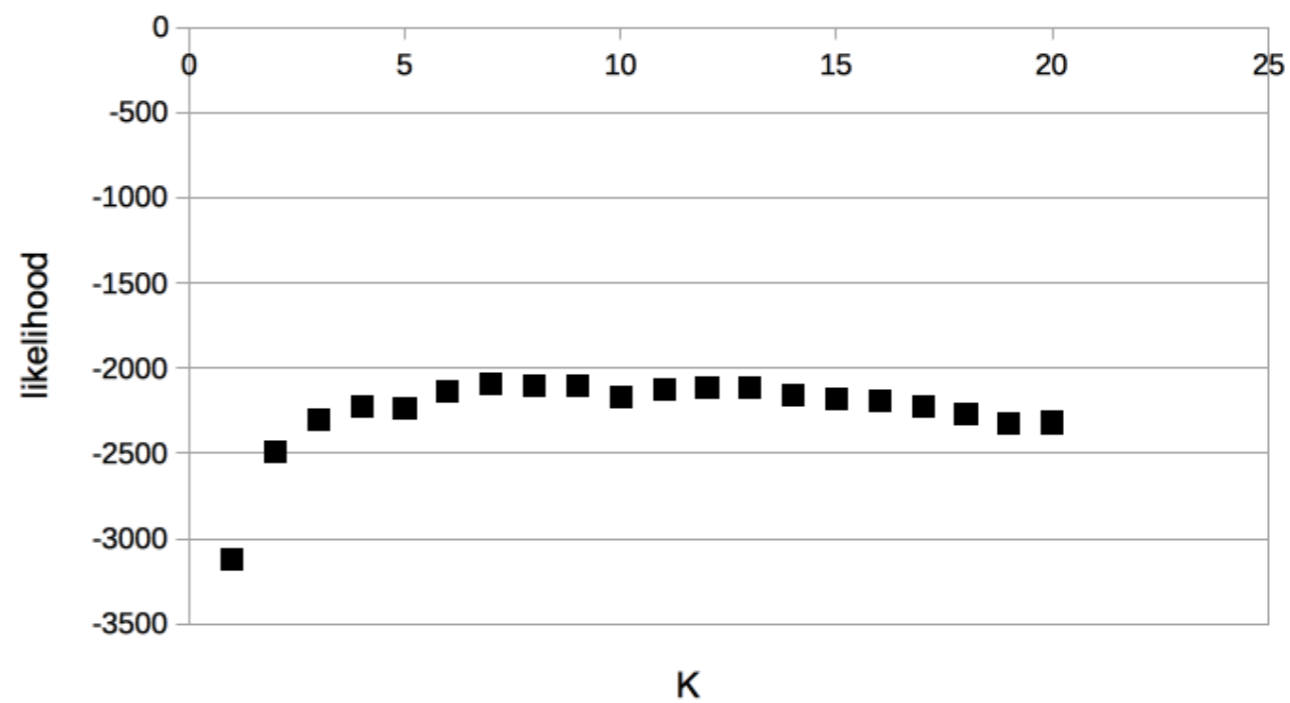

Subset

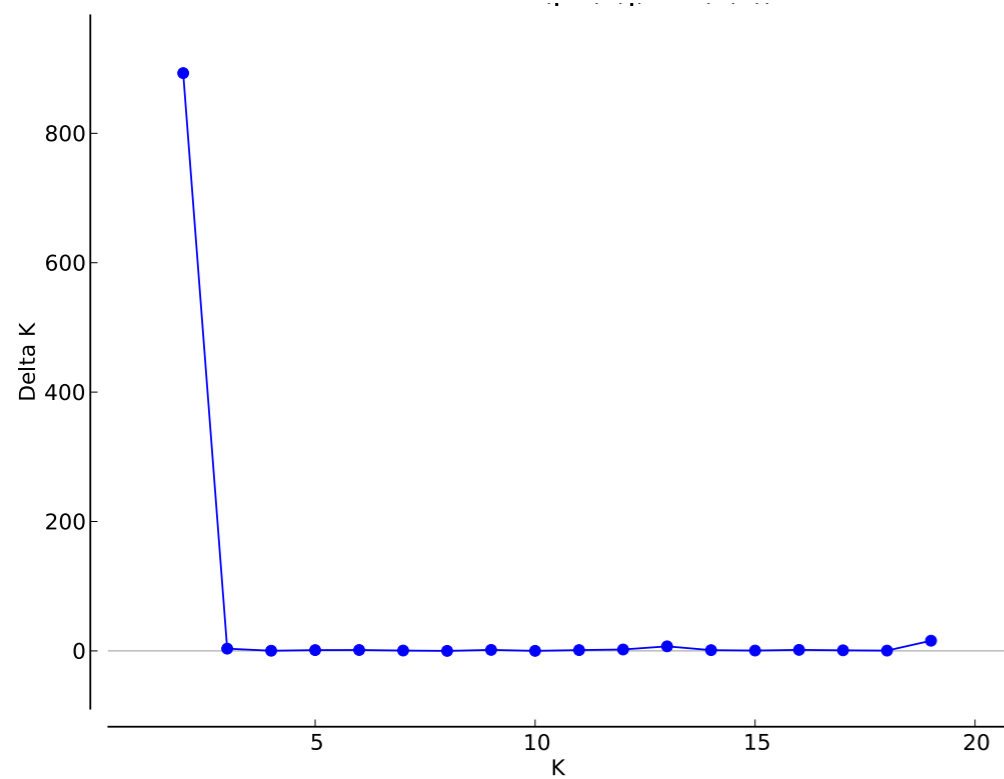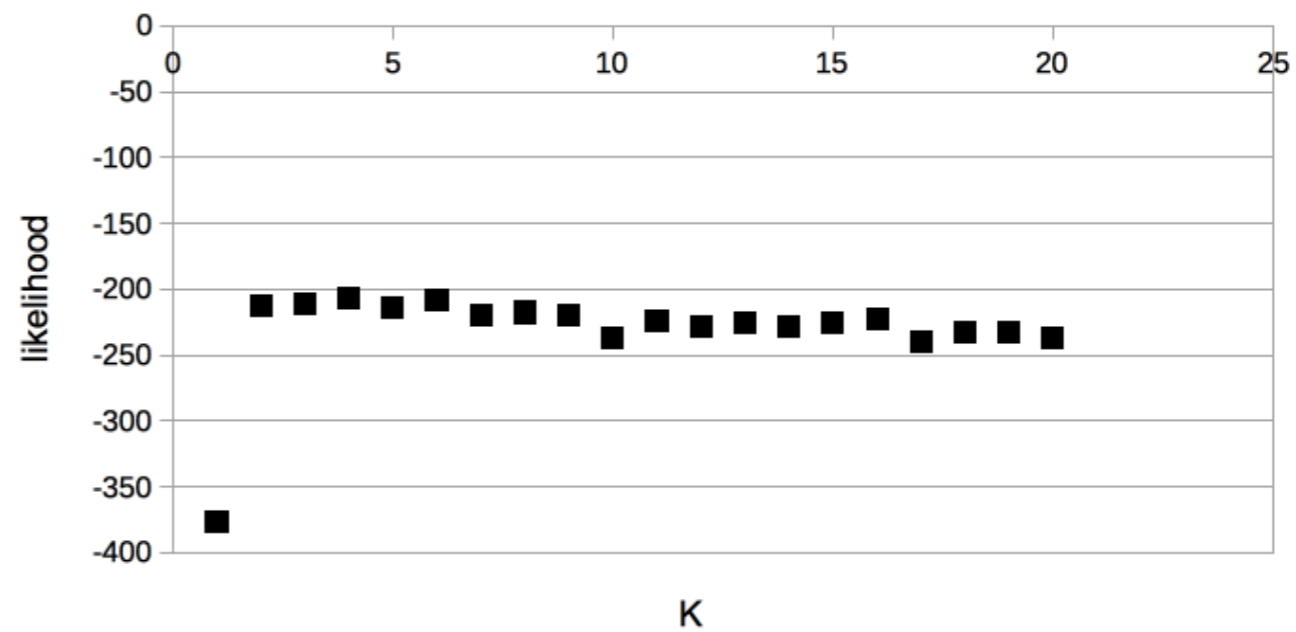

Supplement: Supplementary file 1 [file EVA-10-577-s001.pdf]

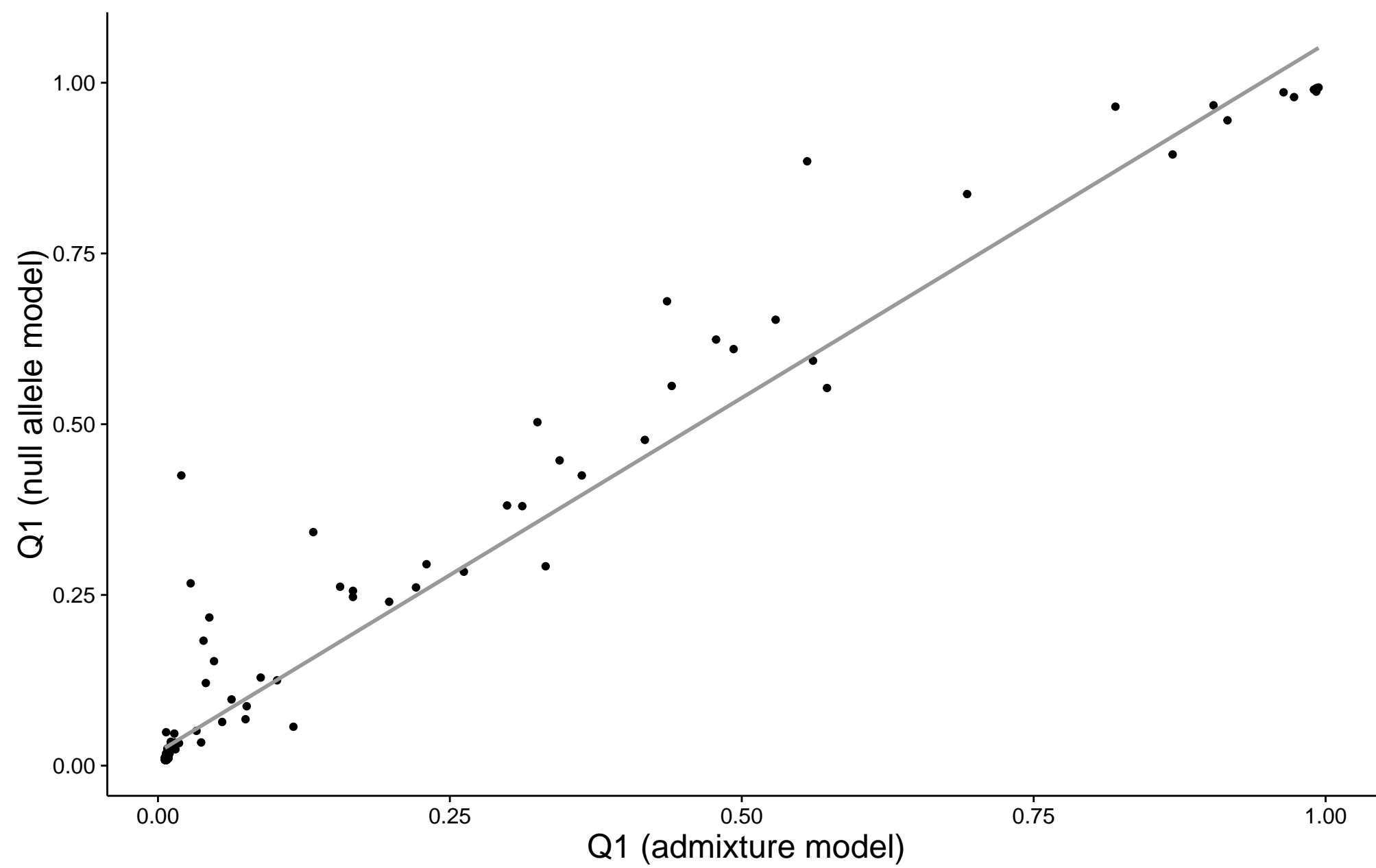

Supplement: Supplementary file 2 [file EVA-10-577-s002.pdf]

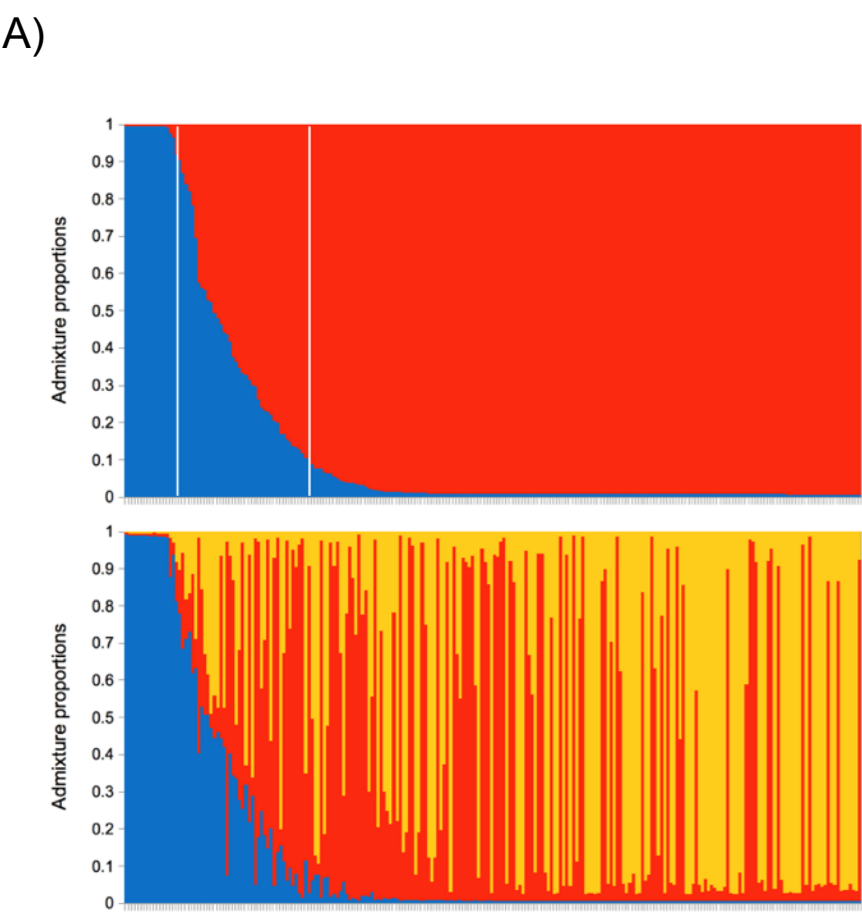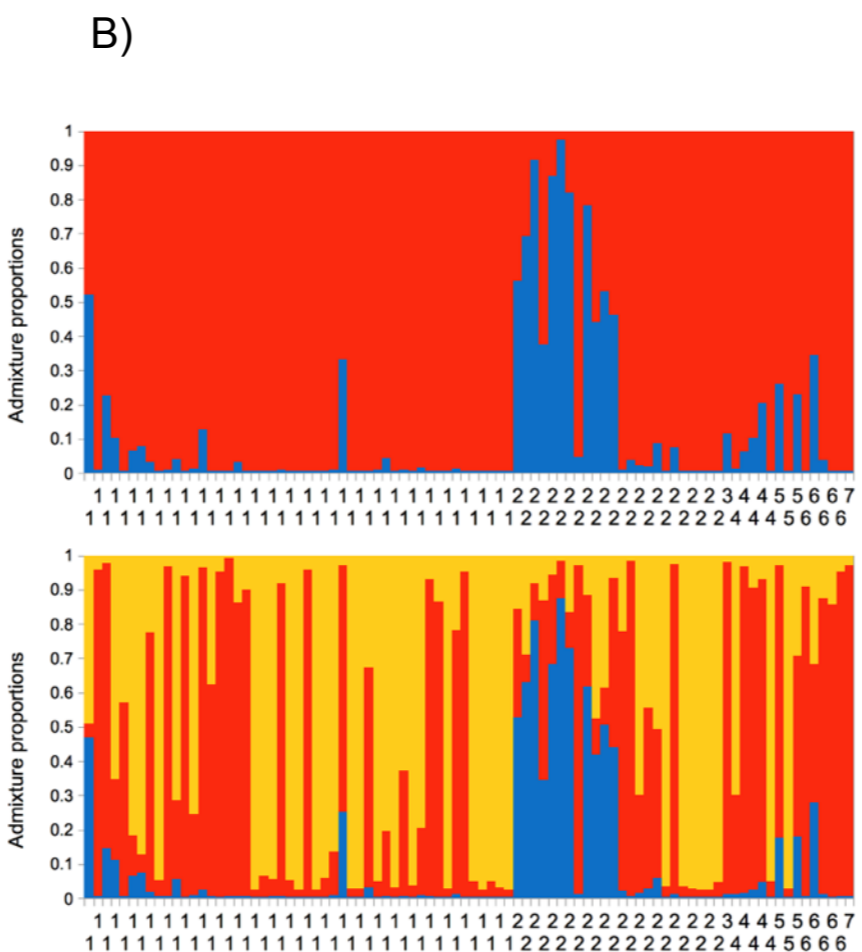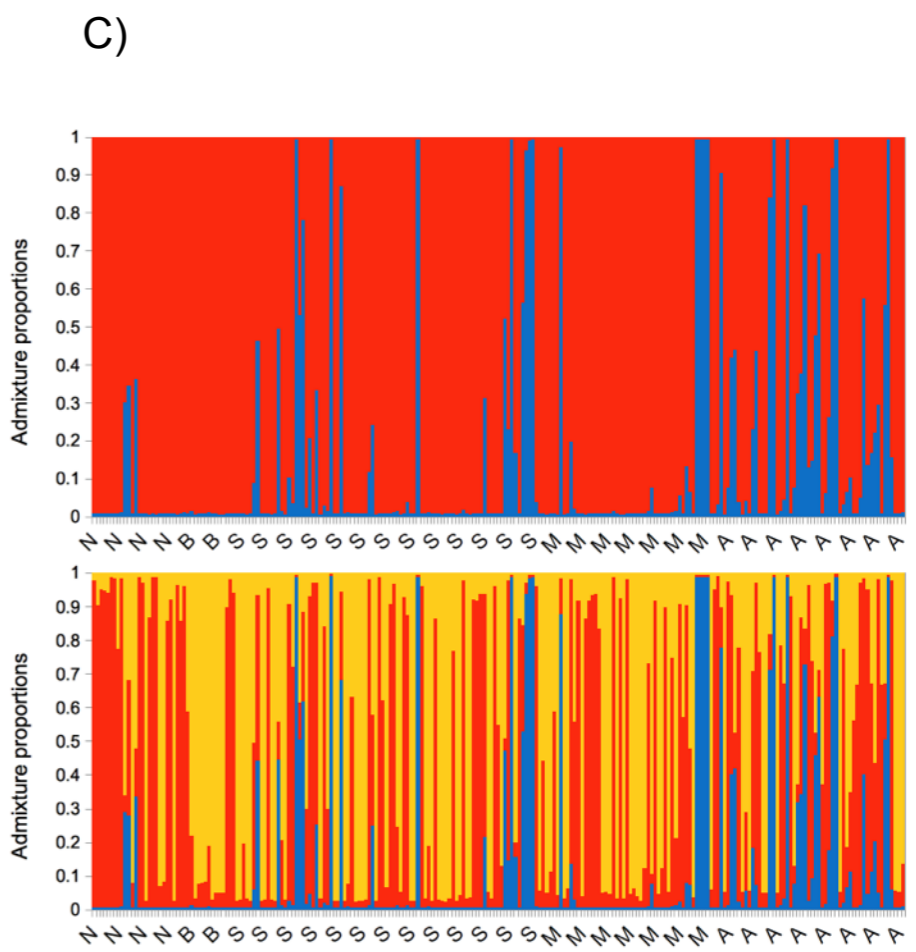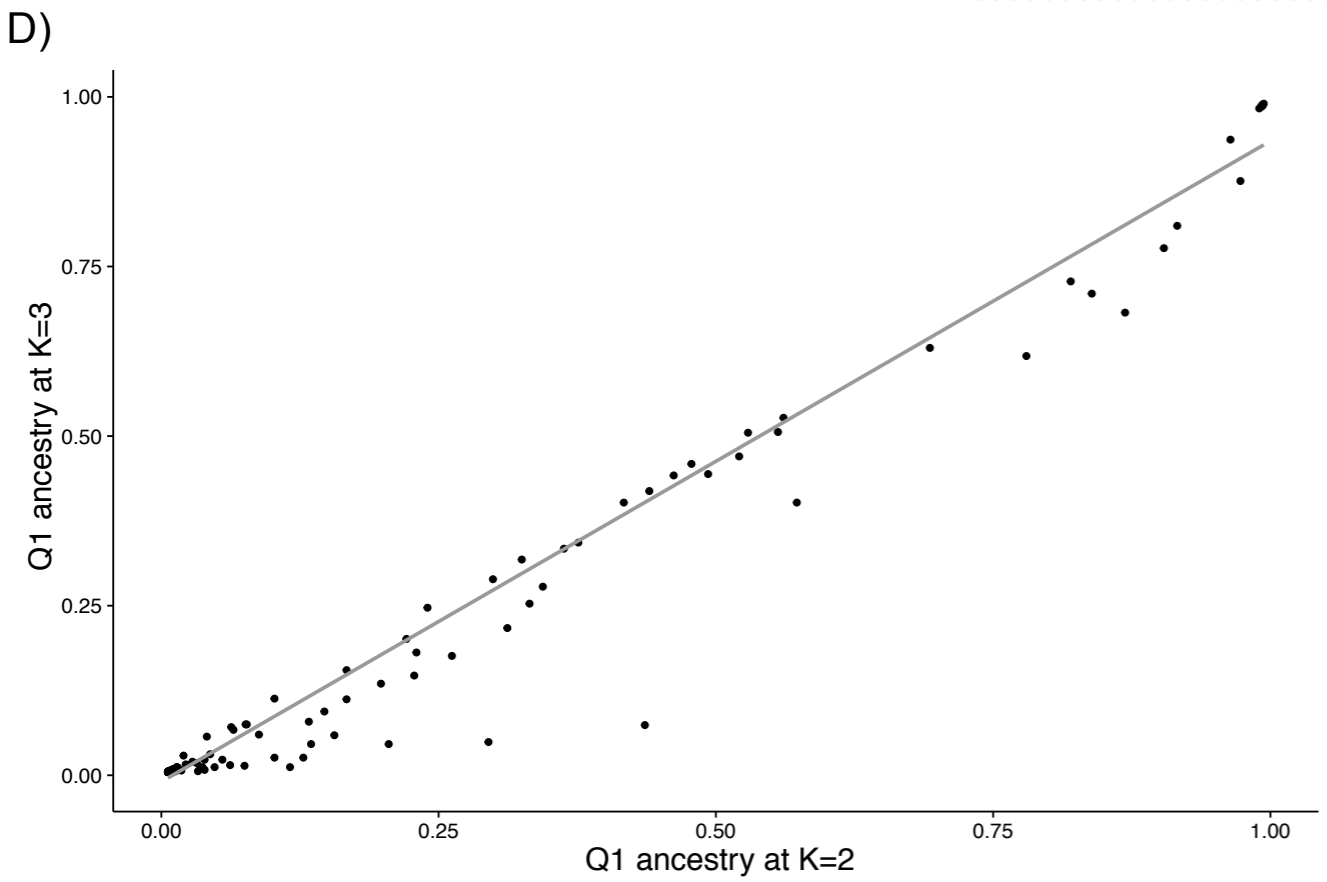

Supplement: Supplementary file 3 [file EVA-10-577-s003.pdf]

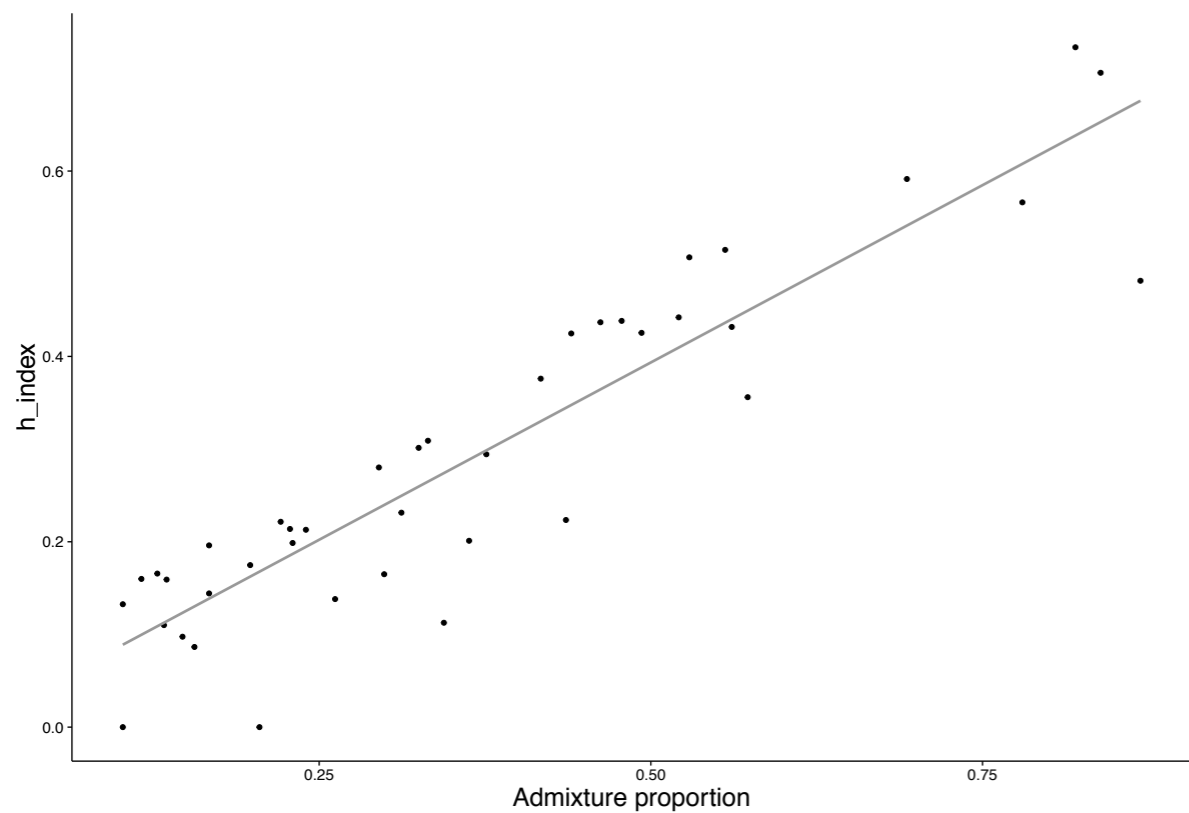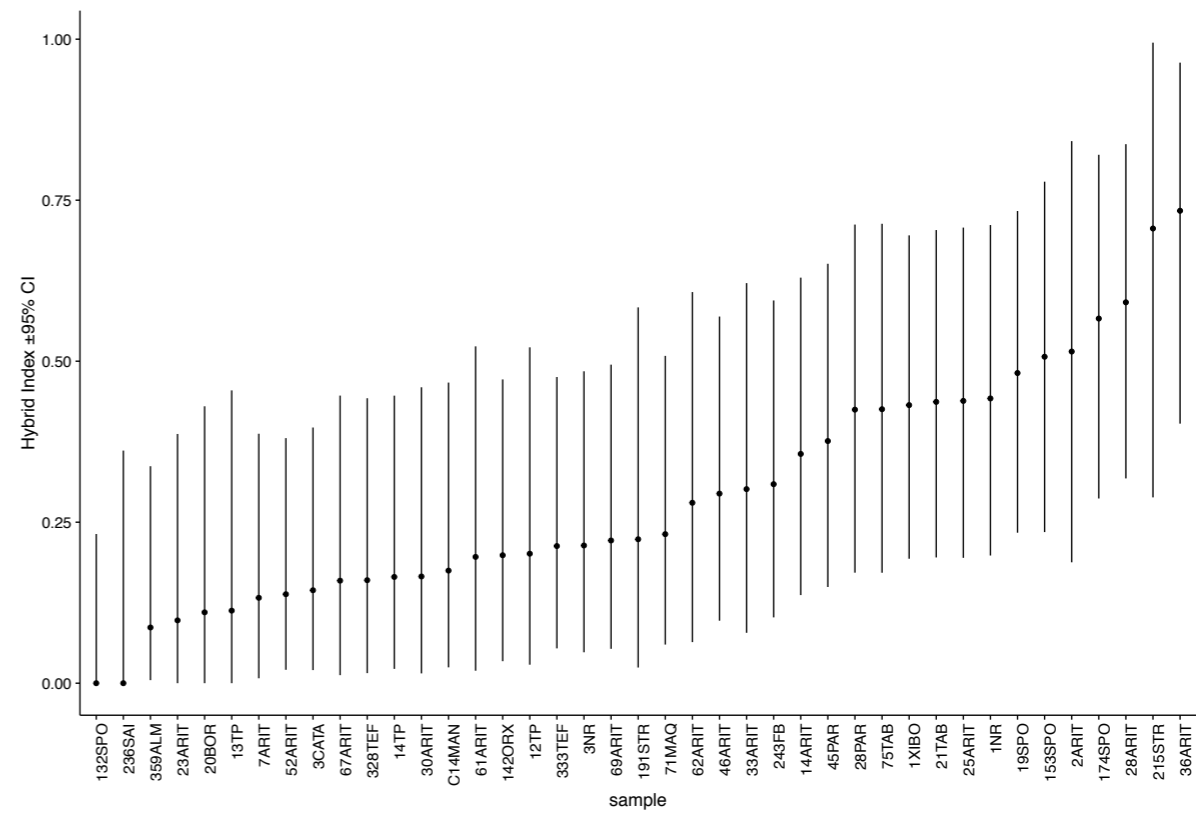

Supplement: Supplementary file 4 [file EVA-10-577-s004.pdf]
