# Supplementary material for: Human management and hybridization shape treegourd fruits in the Brazilian Amazon Basin
Source: Evol Appl. 2017 May 4;10(6):577–89. doi: 10.1111/eva.12474 (PMC5469164; doi:10.1111/eva.12474)
Supplement: Supplementary file 7 [file EVA-10-577-s007.docx]

**Supplementary Materials**

**Tables**Table S1. Passport data of all *Crescentia cujete* and *C. amazonica* collections made in Brazilian Amazonia, with additional information about genetic analyses and morphology. Column A = Collection code; B = Sequencing code; C = Species field identification; D = Local name; E = Latitude; F = Longitude; G = Municipality; H = State; I = Local landscape; J = River basin; K = if analysed for nSSR; L = if analysed for cpSNP; M = Mapped reads from chloroplast sequencing; N = % of missing data in sequence; O = Fruit diameter; P = Fruit shape; Q = Propagation method; R = Nuclear cluster; S = Q1 (%) from Structure analysis; T = Q2 (%) from Structure analysis; U = Chloroplast cluster.

Table S2. List of the 1068 perfect nuclear SSR developed in this study for use with *Crescentia cujete*. The name of the loci, the SSR motif, the number of repeats, the forward and reverse primer sequences, the left primer distance from the SSR (bp), the QDD design and the merged read where they were located, with microsatellites indicated in lowercase font.

Table S3. Eight nuclear SSR primers used to analyze genetic diversity of *Crescentia cujete* and *C. amazonica*. The type and estimated number of SSR repeat motifs, the total number of alleles found in each locus, their size by base pairs (bp) and the primer annealing temperature (Ta), all determined for *C. cujete*.

| Locus | Primer sequence (5'-3') | Repeat  motif | N. alleles | Allele  size (bp) | T_a_ (^o^C) |
| --- | --- | --- | --- | --- | --- |
| Ccuj3 | F:GACACTTGAGAGGGATACAGGG | (TA)^11^ | 9 | 234-270 | 59 |
|  | R:TGTTTGGGTATGGAGTTGTCAGA |  |  |  |  |
| Ccuj4 | F:GGAGAAATAAGGGATACAGATCTACC | (TAT)^8^ | 3 | 193-208 | 59 |
|  | R:TGAAGTGTAAGCCCGAATGACT |  |  |  |  |
| Ccuj7 | F:GGGCTTGGGAAGGAACACAT | (ATA)^10^ | 6 | 165-174 | 59 |
|  | R:ACAACCATGACTCTAGCCTATTACA |  |  |  |  |
| Ccuj8 | F:AACACAACAACGATAATCAAGGG | (AT)^10^ | 11 | 170-190 | 55 |
|  | R:ACAACCATGACTCTAGCCTATTACA |  |  |  |  |
| Ccuj11 | F:ACGTGGCACTGTCCCTATCT | (TAT)^8^ | 3 | 149-160 | 59 |
|  | R:AGTGGAAGATGGGCAGTAGC |  |  |  |  |
| Ccuj13 | F:ACTTGGCTTCTAGCTGTTAGAACT | (TA)^12^ | 8 | 185-203 | 58 |
|  | R:ACAACCATGACTCTAGCCTATTACA |  |  |  |  |
| Ccuj14 | F:GGGAATAATTGGTGGGATTTCAGT | (TC)^11^ | 5 | 153-169 | 55 |
|  | R:ACAACCATGACTCTAGCCTATTACA |  |  |  |  |
| Ccuj15 | F:TAAAGCTCGCAACGACTCGT | (TA)^11^ | 10 | 137-171 | 58 |
|  | R:ACAACCATGACTCTAGCCTATTACA |  |  |  |  |

Table S4. Genetic diversity of *Crescentia cujete* and *C. amazonica* based on 8 nSSR*.* Samples were classified by their levels of admixture according to Structure simulations: pure (ancestry > 0.9), admixed (ancestry < 0.9). *N* = number of samples, *A_r_*= rarefied allele counts, *H_o_* = observed heterozigosity, *H_s_*= expected gene diversity, and mean *F_IS_* (* significant at p < 0.05).

| Species | *C. cujete* | | | | | | *C. amazonica* | | | | | |
| --- | --- | --- | --- | --- | --- | --- | --- | --- | --- | --- | --- | --- |
| Admixture | pure | | | admixed | | | admixed | | | pure | | |
| N | 142 | | | 28 | | | 3 | | | 11 | | |
|  | A_r_ | H_o_ | H_s_ | A_r_ | H_o_ | H_s_ | A_r_ | H_o_ | H_s_ | A_r_ | H_o_ | H_s_ |
| SSR3 | 6.7 | 0.48 | 0.53 | 7.7 | 0.44 | 0.74 | 2.0 | 1.00 | 0.5 | 1.0 | 0.00 | 0.00 |
| SSR4 | 2.0 | 0.54 | 0.43 | 3.0 | 0.67 | 0.56 | 1.5 | 0.50 | 0.38 | 1.0 | 0.00 | 0.00 |
| SSR7 | 4.0 | 0.18 | 0.2 | 4.6 | 0.12 | 0.23 | 1.0 | 0.00 | 0.00 | 1.3 | 0.33 | 0.29 |
| SSR8 | 8.9 | 0.55 | 0.58 | 8.7 | 0.67 | 0.76 | 1.6 | 1.00 | 0.5 | 1.0 | 0.00 | 0.00 |
| SSR11 | 3.0 | 0.03 | 0.06 | 2.0 | 0.33 | 0.49 | 1.3 | 0.33 | 0.28 | 1.0 | 0.00 | 0.00 |
| SSR13 | 6.0 | 0.08 | 0.37 | 7.0 | 0.18 | 0.68 | 1.0 | 0.00 | 0.00 | 1.0 | 0.00 | 0.00 |
| SSR14 | 2.0 | 0.01 | 0.02 | 4.0 | 0.21 | 0.60 | 1.6 | 1.00 | 0.5 | 1.5 | 0.10 | 0.48 |
| SSR15 | 6.6 | 0.17 | 0.27 | 6.4 | 0.54 | 0.59 | 1.6 | 1.00 | 0.5 | 1.0 | 0.00 | 0.00 |
| Mean | 4.9 | 0.25 | 0.31 | 5.4 | 0.39 | 0.58 | 1.4 | 0.60 | 0.33 | 1.1 | 0.05 | 0.09 |
| *F_IS_* |  | 0.19 |  |  |  |  |  |  |  |  | 0.44^*^ |  |
