# Supplementary material for: Human management and hybridization shape treegourd fruits in the Brazilian Amazon Basin
Source: Evol Appl. 2017 May 4;10(6):577–89. doi: 10.1111/eva.12474 (PMC5469164; doi:10.1111/eva.12474)
Supplement: Supplementary file 8 [file EVA-10-577-s008.docx]

**Supplementary Materials**

**Figures**

Figure S1. Estimation of the number of *Crescentia cujete* and *C. amazonica* population clusters identified by Structure simulations based on 8 nSSR and the ad hoc ΔK of Evanno *et al.* (2005). Above. The most likely number of groups (K) using the total sample (N = 234). Below. A randomly chosen subset of the *C. cujete* sample (N = 14) equivalent to the *C. amazonica* sample (N = 14). For each individual, the proportion of admixture obtained from the subset run was compared with the proportion obtained from whole dataset (r^2^ = 0.99, p < 10^-15^).

Figure S2. Impact of null alleles on admixture inference following Falush et al. (2007). For each individual of *Crescentia cujete* and *C. amazonica* with missing data, these were substituted with null homozygotes and the admixture proportions obtained using the Structure specifications for the admixture model were compared to those using the null allele model (r^2^= 0.96, p < 10^-15^).

Figure S3. Comparison of Structure analysis at K = 2 and K = 3 of treegourd samples collected in Brazilian Amazonia using 8 nSSR. A) Above. The full data set (N = 234) at K = 2. The y-axis shows the % of assignment to the groups (red - *Crescentiacujete* and blue - *C. amazonica*). Samples were ordered according to their level of admixture: admixed if > 0.1, hybrids if 0.4 to 0.6, pure if > 0.9 of assignment to the group. Below. The full data set at K = 3. *Crescentia cujete* is divided into red and yellow groups. Middle. B) Within each fruit type (from 1 to 7), samples were arranged by increasing fruit size from left to right (N = 88). Above – K = 2; below – K = 3. C) Samples were arranged according to their geographical location along the main rivers: The Negro (N), Solimões (S) and Amazonas (A) Rivers are ordered West to East; the Branco (B) River is ordered North to South; the Madeira (M) River is ordered South to North. Above – K = 2; below – K = 3. D) The proportion of the blue assignment (*C. amazonica*) (Q1) is similar using K = 2 or K = 3 (r^2^ = 0.98, p < 10^-15^).

Figure S4. Hybridization index (h_index) estimated by Introgress 1.2.3 (Gompert & Buerkle, 2010). Left - relationship between the hybridization index and the proportion of *Crescentia amazonica* ancestry (Q1 < 0.9) of the admixed and hybrid samples (N = 41, see Table 1) obtained by Structure based on 8 nSSR at K = 2 (r^2^ = 0.83, p < 10^-15^). Right - the 95 % confidence intervals of each hybrid sample. The names of samples along the x-axis are equivalent to their passport (Table S1).
